# Mitogenome of *Nasimyia megacephala* Yang & Yang, 2010 (Diptera: Stratiomyidae) and its phylogenetic implications

**DOI:** 10.1080/23802359.2021.1886887

**Published:** 2021-03-17

**Authors:** Kai Hu, Zaihua Yang

**Affiliations:** Guizhou Academy of Forestry, Guiyang, P. R. China

**Keywords:** Mitochondrial genome, phylogenetic analysis, Pachygastrinae, *Nasimyia*

## Abstract

The complete mitogenome of *Nasimyia megacephala* Yang & Yang, 2010 (Stratiomyidae: Pachygastrinae) successfully sequenced. The mitogenome of *N. megacephala* is a circular DNA molecule of 16,069 bp in length with 72% AT content, consisting of 13 PCGs, two rRNAs, 22 tRNAs, and a large control region. The gene order is consistent with other dipteran mitogenomes. The phylogenetic tree was reconstructed using maximum likelihood analysis, and the topology revealed that Pachygastrinae is not a monophyletic group.

The genus *Nasimyia* was erected by Yang and Yang in 2010 (Yang and Yang 2010). It is a small genus of the subfamily Pachygastrinae of Stratiomyidae with four species described (Yang et al. 2013), and all four species were recorded in China. *Nasimyia megacephala* is the type species of *Nasimyia*. Here, we analyzed the mitogenome of *N. megacephala* to provide insights into the phylogenetic relationship of Stratiomyidae.

Adult individuals of *N. megacephala* (GZAF-2020-DS1442) were obtained in Luodian County (E106.7917, N25.4075), Guizhou province, China in May 2020, and were stored in Insect Herbarium of Guizhou Academy of Forestry (URL, Zaihua Yang and yangzaihua008@126.com), Guiyang. Total genomic DNA was extracted from the specimen’s thoracic muscle using the DNeasy Blood & Tissue Kits (Qiagen, Valencia, CA). Genomic DNA was sequenced by the high-throughput Illumina Hiseq 4000 platform. Next, raw data assembled using NOVOPlasty v2.7.0 (Dierckxsens et al. 2017) with *cox1* sequence from *Nemotelus notatus* (GenBank accession no. MT584142) as the initial seed. Finally, the assembled data was annotated by MITOZ v1.04 (Meng et al. 2019).

The mitogenome of *N. megacephala* with a length of 16,069 bp was submitted to GenBank (Genbank accession no. MW115118). All of the 37 typical animal mitochondrial genes (13 PCGs, 22 tRNAs, and two rRNAs) and a control region were identified. All genes display the same order as the putative ancestral arrangement of insects (Clary and Wolstenholme 1985). The AT content of the mitogenome is 72% (37.5% A, 17.7% C, 10.2% G, 34.5% T), indicating significant AT bias. Thirteen PCGs were found to be 11,193 bp in size, accounting for 69.7% of the entire mitogenome. In 13 PCGs, *cox1* starts with TCG, while *atp6*, *atp8*, *cox2*, *cox3*, *cytb*, *nad1*, *nad2*, *nad3*, *nad4L*, *nad4*, *nad5*, and *nad6* share typical start codon ATT/G. Furthermore, all PCGs use TAA/G as the termination codon. The newly-sequenced mitogenome has the complete 22 tRNA genes, ranging from 64 to 72 bp in length. The non-coding control region was detected between *rrnS* and *trnI* is 1,205 bp long with an AT content of 86.1%.

For nucleotide sequence data of 13 PCGs, the best partitioning scheme and nucleotide substitution model for maximum likelihood (ML) phylogenetic analyses were determined with PartitionFinder2 (TIM + I + G for Subset1 (*atp6* and *nad3*), GTR + I + G for Subset2 (*atp8*, *nad2*, and *nad6*), Subset3 (*cox1*, *cox2*, *cox3*, and *cytb*), and Subset4 (*nad1*, *nad4L*, *nad4*, and *nad5*)) (Lanfear et al. 2017). The phylogenetic tree was reconstructed using IQ‒TREE v1.6.3 (Nguyen et al. 2015) with ultrafast bootstrap (UFB) approximation approach (Figure 1) (Minh et al. 2013). Bootstrap support (BS) values were calculated with 10,000 replicates. The phylogenetic relationships among Brachycera is recovered as ((Tabanidae + Athericidae) + (Rhagionidae + ((Xylophagidae + Nemestrinidae) + (Asilidae + (Stratiomyidae + Xylomyidae))))), which is similar to previously published phylogenies (Zhou et al. 2017; Ding and Yang 2020). But our analyses include more taxon sample. Besides, the phylogenetic tree also shows that each family is the monophyletic group with high support (BS = 100). In Stratiomyidae, the monophyly of Pachygastrinae (including *Nasimyia megacephala*, *Parastratiosphecomyia szechuanensis*, and *Tinda javana* in this study) is not supported.

**Figure 1.**
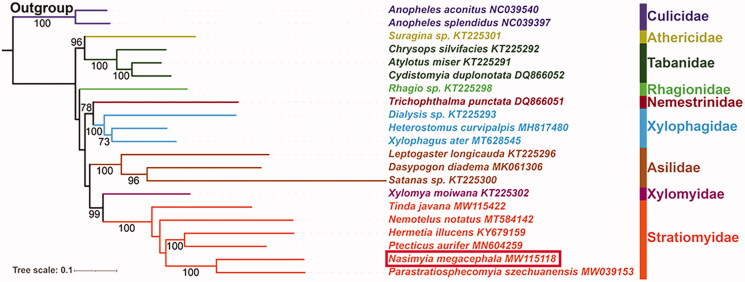
Maximum likelihood phylogenetic tree for Stratiomyidae based on the nucleotide sequence data of 13 PCGs from *N. megacephala* and other 20 species belonging to nine related families of Diptera. The number on each node indicates bootstrap support value (BS). And BS <50 are not presented.

## Data Availability

The genome sequence data that support the findings of this study are openly available in GenBank of NCBI at (https://www.ncbi.nlm.nih.gov/) under the accession no. MW115118. The associated BioProject, SRA, and Bio-Sample numbers are PRJNA693466, SRR13498970, and SAMN17388037, respectively.
